# Bunyaviruses are common in male and female *Ixodes scapularis* ticks in central Pennsylvania

**DOI:** 10.7717/peerj.2324

**Published:** 2016-08-11

**Authors:** Joyce M. Sakamoto, Terry Fei Fan Ng, Yasutsugu Suzuki, Hitoshi Tsujimoto, Xutao Deng, Eric Delwart, Jason L. Rasgon

**Affiliations:** 1 Center for Infectious Disease Dynamics, Pennsylvania State University, University Park, PA, United States; 2 Department of Entomology, Pennsylvania State University, University Park, PA, United States; 3 Molecular Virology, Blood Systems Research Institute, San Francisco, California, United States; 4 Division of Viral Diseases, Centers for Disease Control and Prevention, Atlanta, Georgia, United States; 5 Department of Virology, Institute Pasteur, Paris, France; 6 The Huck Institutes of the Life Sciences, Pennsylvania State University, University Park, PA, United States; 7 Department of Biology, New Mexico State University, Las Cruces, NM, United States; 8 Department of Laboratory Medicine, Blood Systems Research Institute, San Francisco, CA, USA; 9 University of California, San Francisco, CA, USA

**Keywords:** Tick, Virus, Metagenomics, Vector-borne pathogen

## Abstract

The blacklegged tick *Ixodes scapularis* is widely distributed in the United States and transmits multiple pathogens to humans, wildlife and domestic animals. Recently, several novel viruses in the family Bunyaviridae (South Bay virus (SBV) and Blacklegged tick phlebovirus (BTPV)) were identified infecting female *I. scapularis* ticks collected in New York State. We used metagenomic sequencing to investigate the distribution of viruses infecting male and female *I. scapularis* ticks collected in Centre County, Pennsylvania. We identified both SBV and BTPV in both male and female ticks from all collection locations. The role of male *I. scapularis* in pathogen epidemiology has been overlooked because they rarely bite and are not considered important pathogen vectors. However, males may act as reservoirs for pathogens that can then be transmitted to females during mating. Our data highlight the importance of examining all potential avenues of pathogen maintenance and transmission throughout the vector-pathogen life cycle in order to understand the epidemiology of tick-borne pathogens.

## Introduction

The blacklegged tick *Ixodes scapularis* is widely distributed in the United States (Sakamoto, Goddard & Rasgon, 2014) and transmits multiple zoonotic pathogens including *Borrelia burgdorferi* (the agent of Lyme disease (LD)), *Anaplasma phagocytophilum* (the agent of human anaplasmosis), *Babesia microti* (the agent of human babesiosis), Deer Tick Virus/Powassan virus (two closely related tick-borne flaviviruses that cause encephalitis), and potentially nematodes (Zhang, Norris & Rasgon, 2011; Namrata et al., 2014; Henning et al., 2016; Diuk-Wasser, Vannier & Krause, 2016). Epidemiologically, *I. scapularis* nymphs are the most important stage of pathogen transmission to humans because they are more difficult to detect and remove prior to the transmission event (Diuk-Wasser, Vannier & Krause, 2016). Adult *I. scapularis* females are also important in transmission, both directly and by producing new offspring that can subsequently maintain the transmission cycle. Conversely, male *I. scapularis* are not as well studied in relation to *I. scapularis* pathogen epidemiology because they rarely bite and are not considered important pathogen vectors (De Meeûs, Lorimier & Renaud, 2004). However, while biting is uncommon, it does occur and represents an underexplored avenue of pathogen transmission to humans. In addition, some lab studies have shown that male ticks can sexually transfer pathogens to females, suggesting that males could potentially act as reservoirs (Plowright, Perry & Greig, 1974; Hayes, Burgdorfer & Aeschlimann, 1980; Chunikhin et al., 1983; Gonzalez et al., 1992; Alekseev et al., 1999) and may contribute to the epidemiology of pathogens in unexpected ways.

The use of massively parallel sequencing technology has been shown to be very effective in discovery of novel (even unculturable) microbes. Multiple metagenomics studies have recently been published describing known and novel viral sequences from a diverse array of arthropods (e.g. Ng et al., 2011; Tokarz et al., 2014; Xia et al., 2015; Temmam et al., 2015). In one study of mosquito viromes, nearly 50% of approximately 500,000 viral sequences were unidentified (Ng et al., 2011). The rich data set generated from viral sequences purified from mosquitoes’ revealed novel viruses related to those that infect animals, plants, insects, and bacteria (Ng et al., 2011). Virome studies have been conducted in multiple tick species (Tokarz et al., 2014; Xia et al., 2015; Temmam et al., 2015). Tokarz et al. (2014) recently used virome sequencing to detect and identify multiple novel viruses in female *Amblyomma americanum*, *Dermacentor variabilis*, and *I. scapularis* ticks from New York. In addition to the previously identified Powassan virus, they identified several novel bunyaviruses in the genera *Nairovirus* (South Bay virus (SBV)) and *Phlebovirus* (blacklegged tick phlebovirus (BTPV)). These viruses were highly divergent from previously identified tick-borne bunyaviruses (Swei et al., 2013; Tokarz et al., 2014).

In this study, we used metagenomic sequencing to examine the occurrence and distribution of viruses from 18 pools of *I. scapularis* ticks collected in 2014 (nine male, nine female) from multiple populations in and surrounding the State College area of Centre County, Pennsylvania. We identified both SBV and BTPV as the major viruses present in these populations. SBV was identified in all pools and was always predominant, while BPTV was more variable and present at lower levels. These data show that tick-associated bunyaviruses are common in both male and female *I. scapularis* ticks in central Pennsylvania.

## Materials and Methods

### Field collection

**Figure 1:**
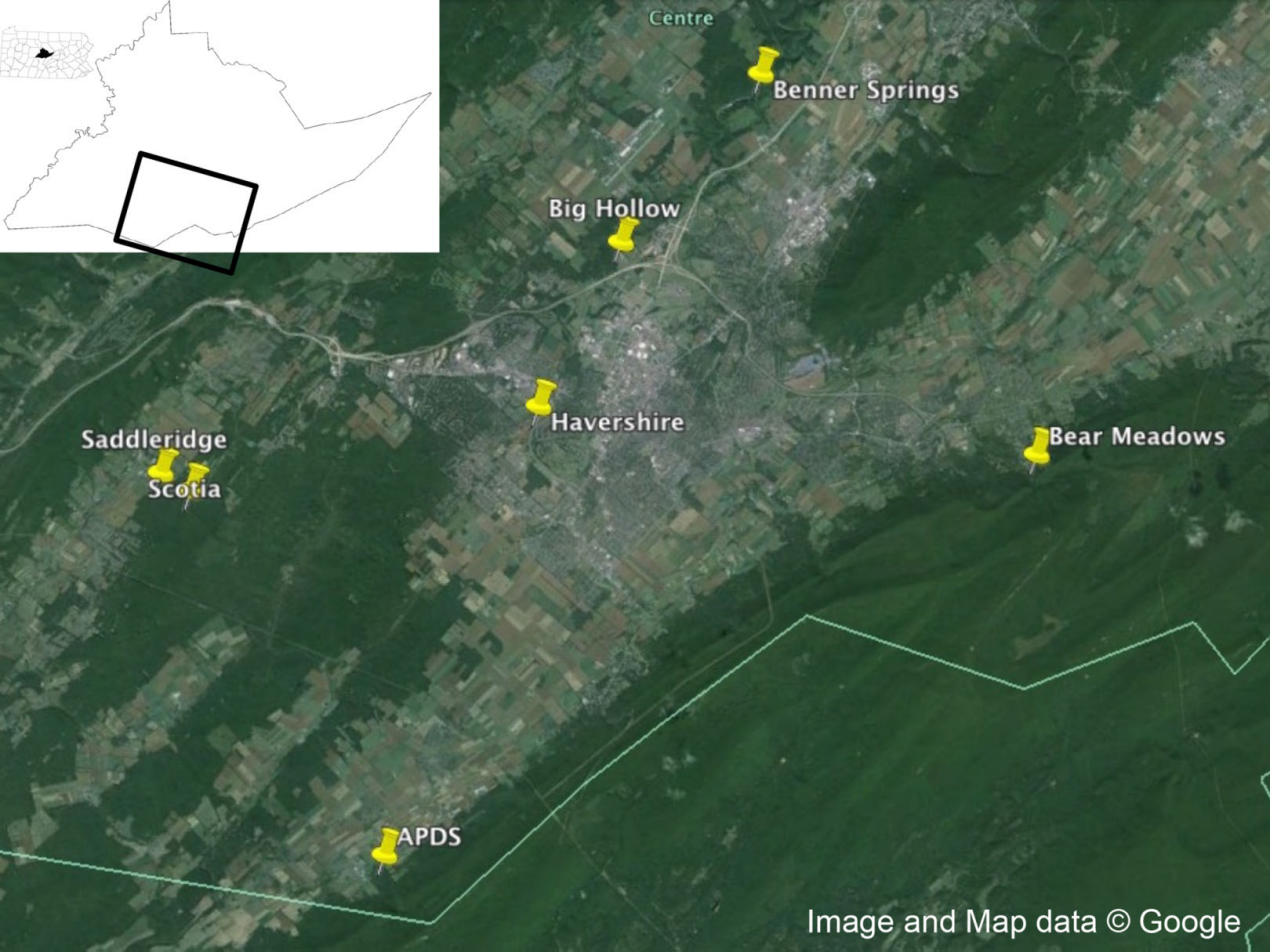
Collection locations. Collection locations in Centre County, Pennsylvania for *Ixodes scapularis* examined in this study. Map data: Google.

Adult male and female *I. scapularis* were collected from Centre County, Pennsylvania in the fall of 2014 using a drag cloth (91.44 × 114.3 cm, Fig. 1). Male and females were separated and ticks stored alive in a 5 ml scintillation vial until returned to the laboratory for visual identification. After identification, live ticks were washed in 70% ethanol for 15 s, then 10% bleach for 1 min, then washed three times in autoclaved, nuclease-free water, and dried on autoclaved filter paper and placed at −80 °C until extraction.

### Homogenization of ticks

Ticks were homogenized individually in 100 μl sterile 1X PBS using sterilized micro-pestles. The homogenate was centrifuged at 1,500 g for 30 min at 4 °C to pellet the tick debris (Ng et al., 2011; Thurber et al., 2009). After centrifugation, 50 μl of the supernatant was pooled with the supernatant from ∼20 other ticks of the same sex and collection site (Table 1). Pooled supernatants were filtered through a 0.45 μm filter.

**Table 1:** Sequencing viral read counts. Collection location, pool information, and Bunyaviridae read counts from metagenomic virome sequencing of central PA *Ixodes scapularis* ticks.

Population	N in pool	Sex	SBV L segment reads	SBV S segment reads	BLTV1 L segment reads	BLTV1 S segment reads	Total SBV reads	Total BLTV1 reads
APDS	16	Female	10,572	53,870	15,090	22	64,442	15,112
APDS	14	Male	86,173	112,175	334	57	198,348	391
Bear Meadows	20	Female	20,100	42,848	5	0	62,948	5
Bear Meadows	20	Male	50,477	60,168	20	0	110,645	20
Benner Springs	20	Female	64,356	125,388	2,031	6	189,744	2,037
Benner Springs	20	Male	319,538	412,178	31	22	731,716	53
Big Hollow	20	Female	10,231	50,032	5	0	60,263	5
Big Hollow	15	Male	132,159	203,425	15,276	2,368	335,584	17,644
Havershire	18	Female	10,684	25,660	136	0	36,344	136
Havershire	15	Male	45,211	57,494	135	0	102,705	135
Saddleridge	20	Female	30,505	38,028	749	0	68,533	749
Saddleridge	20	Female	9,084	15,741	760	0	24,825	760
Saddleridge	20	Male	47,635	74,986	9	152	122,621	161
Saddleridge	20	Male	31,767	46,160	15	2	77,927	17
Scotia	20	Female	144,187	314,304	6,066	408	458,491	6,474
Scotia	20	Female	146,145	288,615	9,425	857	434,760	10,282
Scotia	20	Male	849,797	1,083,252	28,885	9,576	1,933,049	38,461
Scotia	20	Male	148,980	244,248	13,998	1,779	393,228	15,777

### RNase/DNase treatment and viral total nucleic acid extraction

Prior to virion nucleic acid extraction, filtrates were treated with nucleases to remove exogenous nucleic acids (Ng et al., 2011). Each pool was incubated with 14 units Turbo DNase I (Life Technologies/Ambion), 25 units Benzonase (Millipore/Novagen), and 20 units RNase I (Thermo Scientific/Fermentas) for 1.5 h at 37 °C and stopped with DNase stop solution according to the manufacturer’s protocol. Total nucleic acid was extracted immediately after nuclease treatment using the MagMAX viral RNA Isolation purification kit (Life Technologies, Inc.) following the manufacturer’s protocol. Samples were stored at −80 °C until sequenced.

### Next generation sequencing and bioinformatics analysis

Illumina compatible libraries were generated from enriched viral particle preparations using the Nextera XT library prep kit (Illumina, San Diego, CA, USA). Sequencing libraries were normalized using the library quantification kit for Illumina platforms (Kapa Biosystems, Wilmington, MA, USA) prior to sequencing so that the same amount of input material was sequenced for each barcoded library. Next generation sequencing was performed on the MiSeq platform (2 × 250 bp paired-end sequencing). Resulting sequence reads were trimmed, de-duplicated and de novo assembled using a customized NGS pipeline at the Blood Systems Research Institute as described previously (Deng et al., 2015). The assembled contigs and unassembled singlets were compared with a viral proteome database using BLASTx using E-value cutoff 0.01.

### Validation of SBV S segment assembly in individual field-collected ticks

We used the purified virion RNA extracted from pools to generate first-strand cDNA using the ProtoScript® II First Strand cDNA Synthesis Kit (NEB # E6560) following the manufacturer’s guidelines. Confirmation primers (F: AAC-AAG-AGG-TCT-CCG-TTC-CA; R: CTC-GGA-CTT-TTG-GGT-GTG-TG) specific to the SBV S segment assembly were designed using Primer3 (http://frodo.wi.mit.edu) and used to confirm the structure of the viral genome. PCR products cloned, purified, and sequenced in both directions on an ABI 3130/Genetic Analyzer. Sequences were aligned to the viral genome assembly.

### Phylogenetic analysis

We used Maximum Likelihood, implemented in MEGA v. 5.2.2 (Tamura et al., 2011), to phylogenetically compare the full-length aligned L and S segment nucleotide sequences of the bunyaviruses found in this study to complete full-length segments from GenBank. Sequence alignment was performed using ClustalW in MEGA. Tree robustness was assessed through 1,000 bootstrap replications. GenBank numbers included in the phylogenetic analysis are listed in Fig. 3.

## Results

**Figure 2:**
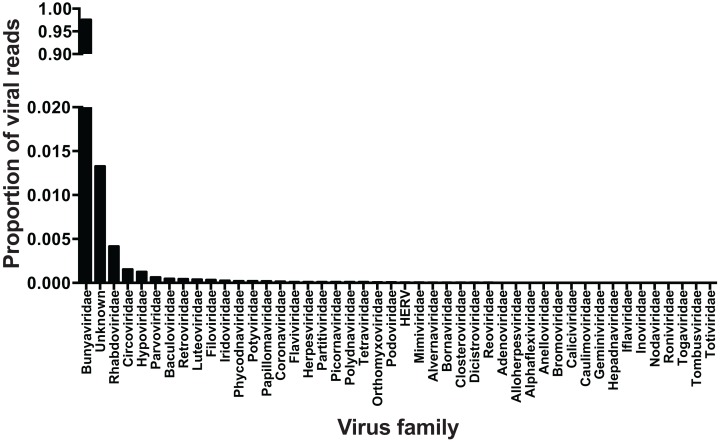
Viral families. Viral families identified in central Pennsylvanian *I. scapularis*. Note break in Y-axis scale.

Metagenomic sequencing of viral cDNA from wild-caught ticks indicated that viral communities in Pennsylvania *I. scapularis* were very non-diverse. Of reads of viral origin (35% of total reads; remainder mapped to the tick host), approximately 98% belonged to members of the family *Bunyaviridae*. An additional ∼1% of reads did not map to any known virus families. The remaining ∼1% of viral sequences mapped to viral families other than *Bunyaviridae*—whether these represent viral infections at low levels or minor contamination during library construction and/or sequencing remains to be determined (Fig. 2). It is clear, however, that if contamination occurred in this study the frequency was very low. Raw sequence data was deposited in the NIH Sequence Read Archive under accession number SRP075634.

**Figure 3:**
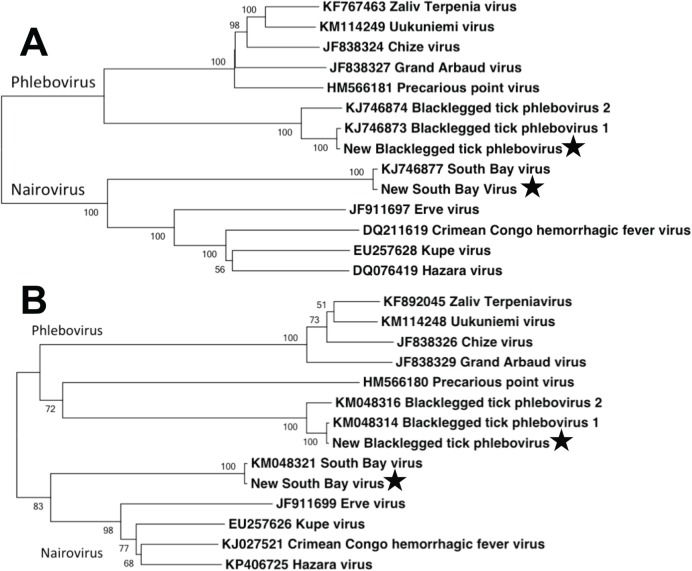
Phylogenetic analysis. Maximum likelihood phylogenetic tree of full-length nairovirus and phlebovirus L segment (A) and S segment (B) nucleotide sequences. GenBank numbers are listed in taxon names. Numbers at tree nodes represent bootstrap support values (1,000 replications). Stars represent sequences obtained in this study.

Within the bunyavirus data, viral sequences belonged to both the genus *Nairovirus* and the genus *Phlebovirus* (*Nairovirus*: 98%, *Phlebovirus*: 2%). The nairovirus SBV was found in all pools, regardless of population or sex. The phlebovirus BTPV was found in all pools but the abundance was highly variable, with very low read counts in several populations (Table 1). We were able to assemble the full-length L and S segments of both SBV and BTPV. There were no significant differences between viral sequences isolated from males vs. females. The obtained L segments matched 98 and 99% to SBV and BTPV1 respectively, while the obtained S segments both matched 98% to SBV and BPTV1 (Tokarz et al., 2015). Results were confirmed by phylogenetic analysis (Fig. 3). Similar to previous studies (Tokarz et al., 2014) we were unable to identify any contigs with homology to the bunyavirus M segment. PCR using specific primers to the SBV S segment resulted in amplification of an approximately 600 bp fragment that mapped 100% to the predicted assembly. SBV and BTPV1 L and S segment sequences were deposited in GenBank under accession numbers KX184198–KX184201.

## Discussion

In terms of disease case numbers, ticks are the most important arthropod pathogen vectors in the United States (Diuk-Wasser, Vannier & Krause, 2016). Most attention has focused on bacterial pathogens such as *Borrelia*, but ticks are important vectors of viral pathogens as well (Swei et al., 2013; Tokarz et al., 2014; Xia et al., 2015; Temmam et al., 2015; Diuk-Wasser, Vannier & Krause, 2016). Tokarz et al. (2014) used next generation sequencing to identify several novel viruses in three species of ticks. In *I. scapularis*, they identified the novel bunyaviruses SBV and BTPV 1 & 2 (two very closely related phleboviruses; Fig. 3). Our results extend the findings of Tokarz et al. (2014), and show that these novel bunyaviruses are present and widespread in *I. scapularis* ticks in central Pennsylvania. We found that these viruses are not only present in females, but are widely distributed at high abundance in male ticks as well.

The role of male *Ixodes scapularis* in pathogen epidemiology has been overlooked, because males often do not take a blood meal. However, males may act as reservoirs for pathogens that can then be transmitted to females during mating (Plowright, Perry & Greig, 1974; Hayes, Burgdorfer & Aeschlimann, 1980; Chunikhin et al., 1983; Gonzalez et al., 1992; Alekseev et al., 1999). These acquired pathogens could then conceivably be transmitted to the vertebrate host during blood feeding or transmitted transovarially to offspring. Our data highlights the importance of examining all potential avenues of pathogen maintenance and transmission throughout the vector-pathogen life cycle in order to understand the epidemiology of these novel tick-borne viruses, and conceptually other pathogens.

## Additional Information and Declarations

### DNA Deposition

The following information was supplied regarding the deposition of DNA sequences: GenBank: KX184198, KX184199, KX184200, KX184201.

### Data Deposition

The following information was supplied regarding data availability: Raw sequence data was deposited in the NIH Sequence Read Archive under accession number SRP075634.

## References

[ref-1] Alekseev AN, Dubinina HV, Rijpkema SGT, Schouls LM (1999). Sexual transmission of *Borrelia garinii* by male *Ixodes persulcatus* ticks (Acari, Ixodidae). Experimental & Applied Acarology.

[ref-2] Chunikhin SP, Stefuktina LF, Korolev MB, Reshetnikov IA, Khozinskaia GA (1983). Sexual transmission of the tick-borne encephalitis virus in ixodid ticks (Ixodidae). Parazitologiia.

[ref-3] De Meeûs T, Lorimier Y, Renaud F (2004). Lyme borreliosis agents and the genetics and sex of their vector, *Ixodes ricinus*. Microbes and Infection.

[ref-4] Deng X, Naccache SN, Ng T, Federman S, Li L, Chiu CY, Delwart EL (2015). An ensemble strategy that significantly improves *de novo* assembly of microbial genomes from metagenomic next-generation sequencing data. Nucleic Acids Research.

[ref-5] Diuk-Wasser MA, Vannier E, Krause PJ (2016). Coinfection by *Ixodes* tick-borne pathogens: ecological, epidemiological, and clinical consequences. Trends in Parasitology.

[ref-6] Gonzalez JP, Camicas JL, Cornet JP, Faye O, Wilson ML (1992). Sexual and transovarian transmission of Crimean-Congo haemorrhagic fever virus in *Hyalomma truncatum* ticks. Research in Virology.

[ref-7] Hayes SF, Burgdorfer W, Aeschlimann A (1980). Sexual transmission of spotted fever group rickettsiae by infected male ticks: detection of rickettsiae in immature spermatozoa of *Ixodes ricinus*. Infection and Immunity.

[ref-8] Henning TC, Orr JM, Smith JD, Arias JR, Rasgon JL, Norris DE (2016). Discovery of filarial nematode DNA in *Amblyomma americanum* in Northern Virginia. Ticks and Tick-Borne Diseases.

[ref-9] Namrata P, Miller JM, Shilpa M, Reddy PR, Bandoski C, Rossi MJ, Sapi E (2014). Filarial nematode infection in *Ixodes scapularis* ticks collected from Southern Connecticut. Veterinary Sciences.

[ref-10] Ng TFF, Willner DL, Lim YW, Schmieder R, Chau B, Nilsson C, Anthony S, Ruan Y, Rohwer F, Breitbart M (2011). Broad surveys of DNA viral diversity obtained through viral metagenomics of mosquitoes. PLoS ONE.

[ref-11] Plowright W, Perry CT, Greig A (1974). Sexual transmission of African swine fever virus in the tick, Ornithodoros moubata porcinus, Walton. Research in Veterinary Science.

[ref-12] Sakamoto JM, Goddard J, Rasgon JL (2014). Population and demographic structure of *Ixodes scapularis* say in the eastern United States. PLoS ONE.

[ref-13] Swei A, Russell BJ, Naccache SN, Kabre B, Veeraraghavan N, Pilgard MA, Johnson BJB, Chiu CY (2013). The genome sequence of Lone Star virus, a highly divergent bunyavirus found in the *Amblyomma americanum* tick. PLoS ONE.

[ref-14] Tamura K, Peterson D, Peterson N, Stecher G, Nei M, Kumar S (2011). MEGA5: molecular evolutionary genetics analysis using maximum likelihood, evolutionary distance, and maximum parsimony methods. Molecular Biology and Evolution.

[ref-15] Temmam S, Monteil-Bouchard S, Sambou M, Aubadie-Ladrix M, Azza S, Decloquement P, Khalil JYB, Baudoin J-P, Jardot P, Robert C, La Scola B, Mediannikov OY, Raoult D, Desnues C (2015). Faustovirus-like asfarvirus in hematophagous biting midges and their vertebrate hosts. Frontiers in Microbiology.

[ref-16] Thurber RV, Haynes M, Breitbart M, Wegley L, Rohwer F (2009). Laboratory procedures to generate viral metagenomes. Nature Protocols.

[ref-17] Tokarz R, Williams SH, Sameroff S, Sanchez Leon M, Jain K, Lipkin WI (2014). Virome analysis of *Amblyomma americanum*, *Dermacentor variabilis*, and *Ixodes scapularis* ticks reveals novel highly divergent vertebrate and invertebrate viruses. Journal of Virology.

[ref-18] Xia H, Hu C, Zhang D, Tang S, Zhang Z, Kou Z, Fan Z, Bente D, Zeng C, Li T (2015). Metagenomic profile of the viral communities in *Rhipicephalus* spp. ticks from Yunnan, China. PLoS ONE.

[ref-19] Zhang X, Norris DE, Rasgon JL (2011). Distribution and molecular characterization of Wolbachia endosymbionts and filarial nematodes in Maryland populations of the lone star tick (*Amblyomma americanum*). FEMS Microbiology Ecology.

